# Exploring Wells-Dawson Clusters Associated With the Small Ribosomal Subunit

**DOI:** 10.3389/fchem.2019.00462

**Published:** 2019-07-05

**Authors:** Debbie C. Crans, Irma Sánchez-Lombardo, Craig C. McLauchlan

**Affiliations:** ^1^Department Chemistry and the Cell and Molecular Biology Program, Colorado State University, Fort Collins, CO, United States; ^2^División Académica de Ciencias Básicas, Universidad Juárez Autónoma de Tabasco, Cunduacán, Mexico; ^3^Department of Chemistry, Illinois State University, Normal, IL, United States

**Keywords:** ribosome, polyoxotungstate, Dawson cluster, H-bonding, protein oxometalate interactions, double Dawson cluster

## Abstract

The polyoxometalate P_2_W_18_${\text{O}}_{62}^{6-}$, the Wells-Dawson cluster, stabilized the ribosome sufficiently for the crystallographers to solve the phase problem and improve the structural resolution. In the following we characterize the interaction of the Wells-Dawson cluster with the ribosome small subunit. There are 14 different P_2_W_18_${\text{O}}_{62}^{6-}$ clusters interacting with the ribosome, and the types of interactions range from one simple residue interaction to complex association of multiple sites including backbone interactions with a Wells-Dawson cluster. Although well-documented that bridging oxygen atoms are the main basic sites on other polyoxometalate interaction with most proteins reported, the W=O groups are the main sites of the Wells-Dawson cluster interacting with the ribosome. Furthermore, the peptide chain backbone on the ribosome host constitutes the main sites that associate with the Wells-Dawson cluster. In this work we investigate the potential of one representative pair of closely-located Wells-Dawson clusters being a genuine Double Wells-Dawson cluster. We found that the Double Wells-Dawson structure on the ribosome is geometrically sound and in line with other Double Wells-Dawson clusters previously observed in the solid state and solution. This information suggests that the Double Wells-Dawson structure on the ribosome is real and contribute to characterization of this particular structure of the ribosome.

## Introduction

**Figure 1:**
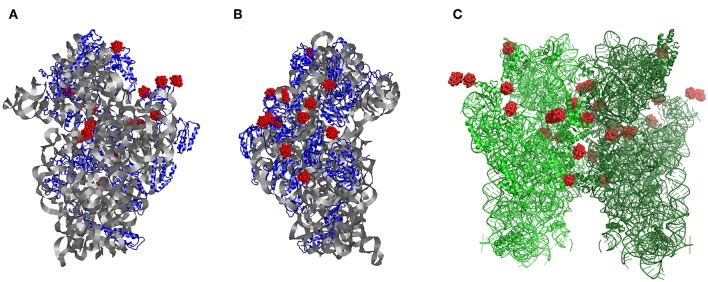
The structure of the small ribosomal subunit from *Thermus thermophilus* (T30S) from the **(A)** front and **(B)** side, respectively. **(C)** Symmetry-generated dimeric form of two ribosomal small subunit structure within their crystals showing each of the P_2_W_18_ sites (red). Modified with permission from Bashan and Yonath (2008).

**Figure 2:**
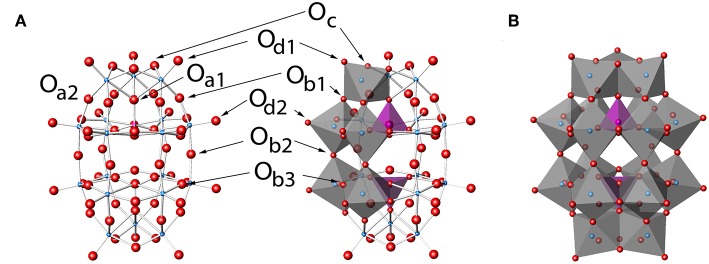
P_2_W_18_, P_2_W_18_${\text{O}}_{62}^{6-}$ in **(A)** ball-and-stick and **(B)** polyhedral representations. Labels in **(A)** show the eight unique types of oxygen atoms and follow a modification to the naming conventions of Keggin clusters (Janik et al., 2003). Darker and lighter polyhedra in **(B)** represent the cap and belt types of tungsten sites, respectively. The Wells-Dawson structure by Kato et al. (2013) with refcode RIBFUF is the structure with the lowest *R*_1_ value reported (Kato et al., 2013) and is used in this work as a reference Wells-Dawson structure.

Polyoxomoetalates (POMs; Wu, 1920; Dawson, 1953; Pope, 1976; Acerete et al., 1979b) have been used for many applications including being a selective and effective inhibitor of enzymes (Stephan et al., 2013), such as ecto-nucleotide pyrophosphatases/phosphodiesterases (NPPs; Lee et al., 2015), and as an artificial proteases (Stroobants et al., 2013), as nanocages for heteroanions (Zheng et al., 2015), and effective in catalysis (Wang and Yang, 2015). POMs have also been found to facilitate X-ray structure analysis of proteins and have been used for solving the structure of proteins such as the small subunit of the ribosome (Janell et al., 2001; Bashan and Yonath, 2008; Yonath, 2009). Proteins are synthesized in an organelle referred to as the ribosome, located in the endosomal reticulum. The large ribosomal subunit contains over 50 different proteins but consists primarily of RNA (over 60%). Understanding the structure for this RNA-protein complex became a goal for many biologists, biochemists, and bioinorganic scientists, considering the importance of this RNA-protein complex for cellular growth. Scientists from several groups were working on solving the X-ray structure for the ribosome, and the successes of three groups in solving the structures of the bacterial small subunit (30S), large subunit (50S), and complete ribosome (70S) structures led to the 2009 Nobel prize to Venkatraman Ramakrishnan (Wimberly et al., 2000), Thomas A. Steitz (Ban et al., 1998), and Ada Yonath (Thygesen et al., 1996; Tocilj et al., 1999). Because of the formidable challenge, the process is still ongoing aiming to determine the structures of ribosomes from different organisms as well as from cells under pressure, so that details can be observed which were not previously accessible. The ribosomal structure has been refined through incremental progress over the past decade by small improvements to overall structure (Figure 1). That is, the co-ordinates are fine-tuned by solving many crystals of interacting species, such as antibiotics, and phasing agents, including the polyoxometalates (POMs) such as the polyoxotungstates shown in red in Figure 1. Over the series of structures of ribosomal small subunit from the extremophile *Thermus thermophilus* (T30S) by Yonath et al. (Thygesen et al., 1996; Tocilj et al., 1999; Weinstein et al., 1999; Schluenzen et al., 2000; Janell et al., 2001; Auerbach-Nevo et al., 2005; Bashan and Yonath, 2008; Yonath, 2009) the Wells-Dawson cluster, P_2_W_18_${\text{O}}_{62}^{6-}$ (P_2_W_18_), (Figure 2) was used extensively. This approach was accompanied by studies with complementary techniques such as electron cryomicroscopy (Cryo-EM) to yield additional details (Winkler et al., 2017; Brown and Shao, 2018). Combined, such approaches have been used resulting in datasets that reveal improved details and allow for better insights into how the polyoxotungstates are stabilizing the protein (Yonath, unpublished data). This manuscript concerns the specific interactions of the most successful POM used in the early crystallographic studies with the small ribosomal unit, P_2_W_18_.

POM clusters, and heavy metals in general, have been especially important for use in X-ray crystallography by stabilizing the POM-protein complexes and several excellent reviews are available on this topic (Dauter, 2005; Dahms et al., 2013; Bijelic and Rompel, 2015). The interactions between POMs and proteins are also of interest in several other contexts considering the reports of specific protein labeling (Kluger and Alagic, 2004), as well as applications of POMs for treatment of various diseases (Hill et al., 1990; Rhule et al., 1998; Stroobants et al., 2013; Leon et al., 2014; Arefian et al., 2017; Bijelic et al., 2018, 2019). The stabilizing interactions between a range of different POMs with proteins continues to be a topic of interest to the scientific community (Bijelic and Rompel, 2015), because of the rising number of structures being reported containing different classes of POMs and other oxometalates (Bijelic and Rompel, 2015, 2017). For example, reorganization of the peptide structure for the tyrosinase (Mauracher et al., 2014) shows that stabilizing interactions are important and may be a general phenomenon and a useful tool for crystallographers with challenging systems (Zhang et al., 2007; Bošnjaković-Pavlović et al., 2011; Dahms et al., 2013; Bijelic and Rompel, 2015, 2017). The advantage of POMs is the presence of heavy atoms to allow for phasing and anomalous dispersion (Weinstein et al., 1999; Rudenko et al., 2003; Dauter, 2005; Blazevic et al., 2015), but another second advantage is that the anionic nature of POMs, as Rompel and co-workers noted, “could act as a ‘glue’ to connect these otherwise electrostatically repulsive surfaces” (Zhang et al., 2008; Mauracher et al., 2014), a fact which has also been observed for the Ribosome T30S as well (Auerbach-Nevo et al., 2005; Bashan and Yonath, 2008; Yonath, 2009). The fact is that several POMs are included in the kits currently commercially available to life scientists to assist protein crystallization illustrate the effectiveness of these systems (e.g., Jena Biosciences, 2019). Although the improvement in quality of solved protein structures through the use of POMs in co-crystallization has been profound, it is striking that the major success with the ribosomal subunit remained fleeting until the Wells-Dawson POM was employed (*vide infra*), particularly because these POMs have not been used in studies with other proteins.

The Wells-Dawson POM cluster structure shown in Figure 2 was found to be the most effective stabilizing cluster used to begin to solve the phasing problem for the ribosome by the Yonath group (Tocilj et al., 1999). The P_2_W_18_ cluster has long been known to have two unique tungsten sites (Acerete et al., 1979b), namely the belt and the cap (Figure 2B), and was one of the first species ever studied using ^183^W NMR spectroscopy owing to its stability and well-defined structure (Acerete et al., 1979a). The cluster ideally possesses *D*_3h_ symmetry (Contant and Thouvenot, 1993; Vilà-Nadal et al., 2012), although there is little energetic difference between other symmetry options such as *D*_3d_ (Vilà-Nadal et al., 2012). There are eight different oxygen sites in the cluster, some exhibiting differences based on symmetry and some on chemistry (see Figure 2A; Dawson, 1953; Janik et al., 2003; Poblet et al., 2003). Interestingly, this POM has not been reported in any other *deposited* protein-POM structures besides the T30S Ribosome. Based on its structure one can thus anticipate that this oxometalate is particularly useful for different systems because the variety of possible sites on the P_2_W_18_ clusters are likely to interact differently with the protein surface and allow more types of interactions than, for example, the flat Anderson-Evans structure type (e.g., TeW_6_${\text{O}}_{24}^{\text{n}-}$). The question of whether this POM exhibited similar stabilizing effects of other POMs in general is therefore of particular interest to the scientific community and there is a need to be able to examine the protein-oxometalate interactions in various published structures (Bijelic and Rompel, 2015, 2017). However, scientists working on the large protein structures have sometimes used the concept of “form factor,” so that the electron density in these large oxometalates are averaged, and only one central spot of the POM molecules are reported in the newer structures (or no evidence for POM was reported; Ban et al., 1998, 1999)*. For an inorganic chemist interested in the interactions of the POM with the protein or those that wanted to use this complementarity for design of future systems such an approach is very limiting. Specifically, one cannot always simply download the protein structural files and examine the interactions between the oxometalate from the newer X-ray structures because the coordinates deposited are often simplified using form factors leading to incomplete descriptions of the details of the interactions between the oxometalate and the protein*.

Examining the interactions of the metal complexes with proteins is interesting but has several issues, as detailed in previous works (Janell et al., 2001; Crans et al., 2014; McLauchlan et al., 2015; Sanchez Lombardo et al., 2015). Briefly, issues relating to resolution (Weinstein et al., 1999; Rudenko et al., 2003; Dauter, 2005; Blazevic et al., 2015), phasing (Thygesen et al., 1996; Pioletti et al., 2001), and degradation (including photoreduction) (Rich et al., 1998; George et al., 2012) all become of critical importance. Protein X-ray crystallography is thus very different than small molecule crystallography because the protein structure diffraction data are often collected across numerous crystals rather than a single crystal more typical of a small molecule, although techniques have been developed to alleviate this issue in certain cases (Janell et al., 2001; Heras and Martin, 2005). Seeking improvements in the structure, interacting species, such as antibiotics, and phasing agents, including the polyoxotungstates were used to improve the overall structure (*vide infra*). Because the focus of the investigating researchers is on the main structure of the proteins, not the interacting species, it is often the case that the non-protein metal clusters are not even shown or deposited (e.g., Ban et al., 1999). This simplification is in contrast to the inclusion/treatment of the antibiotic species' coordinates, usually because they are deemed potentially more relevant (Pioletti et al., 2001). The lack of deposited coordinates for the metal clusters, then, makes studying protein-metal interactions a challenge in those cases.

The Wells-Dawson polyoxometalate, P_2_W_18_${\text{O}}_{62}^{6-}$ (P_2_W_18_), provided the heavy metal electron density critical to one of the approaches to managing the phase problem for solving the structure of the small ribosomal subunit (Brodersen et al., 2003; Dauter, 2005; Barrier et al., 2009). Since first described by Perutz with hemoglobin (Green et al., 1954), heavy atom derivatization has been employed for many years in solving structures and is well-reviewed as a technique in the literature (Garman and Murray, 2003; Dauter, 2005). In practice, ribosomal protein crystals may be soaked with P_2_W_18_ solutions and large amounts of the P_2_W_18_ remain in the crystal even after washing, helping with diffraction and phasing (Janell et al., 2001). In addition, the Yonath group reported that the P_2_W_18_ POM served to anchor the protein conformation and stabilize the ribosomal proteins in their preparations, although other groups did not observe similar stabilization of the ribosome in their preparations (Wimberly et al., 2000; Clemons et al., 2001). The stabilization led to superior diffraction patterns and to the improved resolution X-ray structure solved by Bashan and Yonath (2008). Incremental progress in resolution was obtained from above 4 to 3.3 Å and below (Thygesen et al., 1996; Tocilj et al., 1999; Weinstein et al., 1999; Schluenzen et al., 2000; Auerbach-Nevo et al., 2005; Bashan and Yonath, 2008; Yonath, 2009). This structure was a vast improvement compared to the low resolution structures investigated earlier with no P_2_W_18_. In addition to providing the increased electron density the P_2_W_18_ caused some significant structural organization in the ribosomal protein subunit S2, (Pioletti et al., 2001; Auerbach-Nevo et al., 2005) which in this unit cell is situated proximal to the crystallographic 2 axis. Because use of P_2_W_18_ allowed improvement in the resolution of the T30S structure in a way not seen using other POMs [even though those POMs are successfully employed in other protein structures (Bijelic and Rompel, 2015)] this led us to examine the nature of the P_2_W_18_ interactions with the biological portions of the structure. In some of the recent ribosome structures, however, the deposited protein structures contain the spherically averaged form-factor (labeled PW) and are represented as a point charge near the protein, as is often employed when non-symmetric heavy metal agents with not-necessarily-specific interactions with the protein are used in this way (Thygesen et al., 1996; Yonath, unpublished data). Therefore, all the detail in the interaction of the POM with the protein is lost, and not accessible to the bioinorganic chemist or other crystallographers wanting to use these types of systems for future crystallization of new proteins.

In the following we use data-mining studies to explore specifically how the Wells-Dawson cluster and a possible Double Wells-Dawson cluster interact with the ribosomal protein. To carry out this analysis we introduce a systematic approach that can be employed while investigating the POM-protein structures deposited in the Protein Data Bank (PDB; Berman et al., 2000). Some of these protein structures include the spherically averaged form-factor (“PW”) and no longer contain the detailed electron density for POM near the protein (Schluenzen et al., 2000). Using the structure of one crystallographically-characterized Wells-Dawson molecule we can complete the model for any incomplete P_2_W_18_ POM-structures on the protein by overlaying a selected Wells-Dawson model structures. As a result, we will be able to examine the structures of the reported POM-ribosome complexes, in which the POM-unit was not reported intact in the PDB. We specifically investigate using data mining whether two closely located P_2_W_18_ POMs, “Double Wells-Dawson,” associated with the ribosome is likely real. These studies provide information on the interaction of the Well-Dawson structure with a protein, and illustrate a strategy to investigate these types of protein structures in which spherically averaged form-factors are used to indicate the location of a POM.

## Experimental

### Files of Ribosomes From Protein Data Bank (PDB)

**Figure 3:**
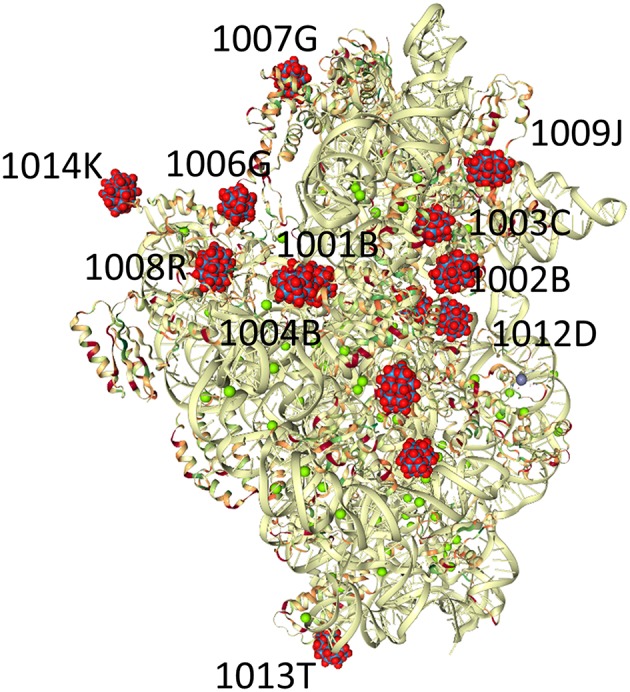
Crystals of the small ribosomal subunits (T30S) were soaked for a few minutes in solutions containing small amounts of the Wells-Dawson tungstate complex (P_2_W_18_, PW_18_ by the authors, WO2 in the PDB). The structure of the complex of the ribosomal subunits with the cluster (PDB Ref code 1I94) is shown. The P_2_W_18_ residues that are described in detail in this manuscript are P_2_W_18_ numbers 1,014 and 1,006 for the discrete clusters and 1,001 and 1,004 for the Double Wells-Dawson cluster (Pioletti et al., 2001).

The structure of the small ribosomal unit has been solved in pieces with incremental progress in a series of works (Thygesen et al., 1996; Tocilj et al., 1999; Weinstein et al., 1999; Pioletti et al., 2001; Auerbach-Nevo et al., 2005; Bashan and Yonath, 2008; Yonath, 2009), culminating in the report of the T30S structure reported in 2000 (Schluenzen et al., 2000) and deposited in the Protein Data Bank (PDB) (Berman et al., 2000) with PDB code 1FKA (resolution 3.3 Å). This original publication (Schluenzen et al., 2000) modeled the electron density of a P_2_W_18_${\text{O}}_{62}^{6-}$ unit at the center of mass of the cluster labeled WO_2_ as the “spherically averaged form-factor” with the label “PW” (Schluenzen et al., 2000). Subsequent work included further inclusion of the clusters in more detail (Pioletti et al., 2001). The original authors used the Crystallography & NMR System (CNS, Brünger et al., 1998) for their refinements. The Protein Data Bank (PDB) defines Wells-Dawson as “WO_2_” and all WO_2_-containing structures were examined and the coordinates downloaded (Table S1). We concentrated our efforts on the best resolution structure for the Ribosome T30S from the PDB, PDB code 1I94 (Figure 3; Pioletti et al., 2001). In examining the interactions of the POMs with T30S, the locations of the POMs must first be established before the adjacent protein residues are identified.

### Selection of Model Wells-Dawson Structure

An analysis of the Cambridge Crystal Structure Database (CSD, November 2017) (Allen, 2002) affords 146 hits with the composition “P_2_W_18_O_62_” as part of a single molecule. The structure of a hydrated lithium salt by Kato et al. (2013) with refcode RIBFUF is the Dawson complex with the lowest *R*_1_ value (Kato et al., 2013), which is taken to correspond to the best model fit in the literature. We used the anion in this Wells-Dawson-containing structure as the archetypical cluster for examination.

### Completing POM Structures Associated With the Ribosome

For structures containing the Wells-Dawson clusters missing atoms or those X-ray structures in which averaged form-factors (PW) are used (Schluenzen et al., 2000), it is not trivial to access the details in the structural interaction of the Wells-Dawson-ribosome complex. That is, oxygen atoms and in some cases W-atoms can be missing and not provide a complete description of the P_2_W_18_ cluster.

When the averaged form-factors are used by the crystallographers there are no details regarding the interaction of the Wells-Dawson cluster associated with the ribosome. However, given the well-defined nature of Wells-Dawson clusters, it seemed reasonable that an overlaid ideal structure could be identified based on known structures and that such constructs would suggest where the missing atoms should be and provide the interactions of the entire cluster with the protein or RNA portions of the ribosome. This is particularly important because the missing oxygen atoms generally are at the surface of the POM and thus likely to engage in H-bonding with the ribosome. This is not possible when a point sphere is used, but it can be minimized when at least a portion of the cluster is present.

## Results

Most publications mentioning the P_2_W_18_ clusters in the T30S structures mention seven P_2_W_18_ sites (e.g., Janell et al., 2001), and there are only seven unique WO2 sites in 1FKA (Schluenzen et al., 2000), however, there appear to be 14 crystallographically-unique locations in 1I94, each half-occupied by a cluster. Experiments showed that more tungsten was present in the crystals than accounted for in the X-ray diffraction studies (Janell et al., 2001), but the choice of 50% occupancy is a reasonable one in the absence of any compelling data to the contrary. It is not surprising that the clusters may not have similar crystallographic occupancy over the entire clusters (Tocilj et al., 1999), and although it could reflect lacunary structures associated with the protein, it could also simply be a statistical averaging. This occupancy is of particular importance in examining the P_2_W_18_ clusters in the T30S structure because, although most of the clusters on the ribosome are spread out over the surface of the minor ribosomal subunit, three pairs of clusters are particularly close together. In 1I94, eight of the Wells-Dawson cluster sites appear discrete, that is there is no other P_2_W_18_ cluster within 10 Å, but six of the clusters appear to be in pairs, much closer to one another. The three pairs of clusters reside at closest interactions of 2.365, 4.364, and 6.297 Å, respectively.

### Three Discrete Wells-Dawson Structures: 1,014, 1,006, and 1,576

The large and stable nature of the clusters made them useful for phasing and even if an entire cluster was not visible in the electron density map, the X-ray crystallographers rationalized that although the resolution of the ribosomal structure is not sufficient to identify all of the clusters atoms, they could take advantage of the well-known structure of the Wells-Dawson cluster and focus the model so that details about the structure “of interest,” namely the ribosome RNA-protein complex, can be extracted. In some structures (e.g., PDB ID 1I94) the clusters are more complete than in others and the deposited coordinates may not even contain any details about the P_2_W_18_, i.e., PDB ID 1FKA. In examining the ribosome structures containing P_2_W_18_, the number of P_2_W_18_ sites is not identical across all five of the deposited T30S structures. In PDB ID 1I96, for instance, several of the P_2_W_18_ clusters found in the others of the series (i.e., 1I94, 1I95, and 1I97) are missing (Figure S1).

**Figure 4:**
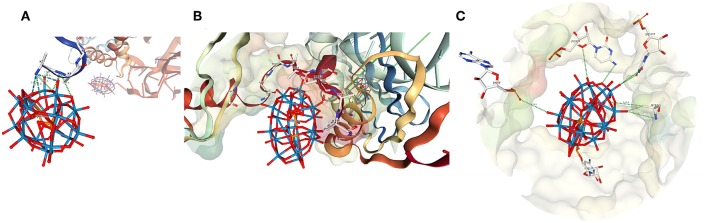
The structure of POMs number **(A)** 1,014 **(B)** 1,006, and **(C)** 1,576 from 1I94. Numbering as shown in Figure 3 (Pioletti et al., 2001).

Cluster 1,014 is off on the periphery of the ribosome surface (Figures 3, 4). This P_2_W_18_ interacts strongly with many interactions with one Lys residue through the W-atoms on the belt of the P_2_W_18_. The location of this Wells-Dawson on the tip of the ribosome unit may seem surprising; this POM, though, serves a key role to organize the different protein units in the crystal (interactions not shown), and interacts with proteins in adjacent unit cells. In the case of cluster 1,006, a very different mode of interaction is observed and close interactions with many peptide parts including strands in the peptide chains G (x 2) and K (x 2 alpha helices) (Figure 4). This cluster has the entire peptide backbone chain wrapped around it showing interaction with the backbone of a number of amino-acids. The peptides involved in this interaction with cluster 1,006 include Ala, Arg, Ala, Tyr, Ala, Tyr, Arg, and Trp.

In this article our focus is on the protein interactions with many of the P_2_W_18_ clusters in 1I94, however, considering the high RNA content, there are also some interactions of some P_2_W_18_ clusters with the RNA as well. For example, cluster 1,576 is one such cluster that interacts with RNA (Figure 4C). Portions of this cluster also interact with a Gln side chain, but there are also interactions between the terminal oxo units and phosphate backbones, ribose oxygen, and oxygen and nitrogen from uracil. Given the prevalence of RNA in the ribosomal subunit and the fact that the interacting RNA units each come from different RNA strands in this case, cluster-RNA interactions certainly help play a part in the stability of these crystals. Further analysis and investigation of such interactions are warranted, but they are not our primary focus here.

### Completing the Wells-Dawson Structures

In some data sets, including some still unpublished, the complete P_2_W_18_ clusters are not present or are only partially found in the electron density difference maps. We had originally considered modeling such a system but instead focused on the publicly available data sets as a starting point. It is impossible to model the spherical point used in 1FKA, but a partially complete P_2_W_18_ cluster would have been possible. An idealized P_2_W_18_ unit (*vide infra*) was placed using the structural overlay feature of *Mercury* (Macrae et al., 2008) and the “define cluster feature” of Crystal Maker allowing us to approximate the missing oxygen-atoms in the P_2_W_18_${\text{O}}_{62}^{6-}$ (Figures 2, 3) for those protein structures where P_2_W_18_ is not fully found.

This structure was used to measure the distances of each amino acid or backbone atom to the oxygen-atoms on the surface of the P_2_W_18_ unit. When present, experimentally-located oxygen atoms were used. Typically, the differences in distances between experimentally-located oxygens vs. idealized oxygens were on the order of 0.010 Å. We anticipate that in 5 years or less, it may even be possible to use a routine program to provide these types of overlays and, therefore, interactions.

### Possible Structures for Double Wells-Dawson Structures

**Figure 5:**
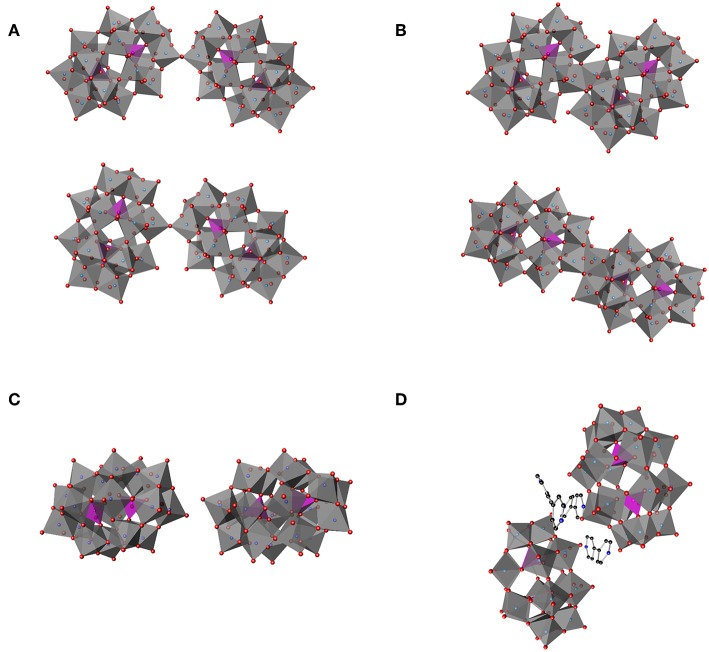
Schematic illustration of several hypothetical double Dawson clusters. **(A)** Double Dawson structures may be connected through a joint oxygen atom on a corner, corner-shared (P_2_W_18_O_61_)_2_O, in a cap-to-cap (top) or belt-to-cap (bottom). **(B)** Two structural possibilities where the Double Dawson structure are connected through the edge, edge-shared (P_2_W_18_O_60_)_2_O_2_, with belt-to-belt (top) and cap-to-cap (bottom). Two full P_2_W_18_ Dawson units can be interacting through **(C)** H-bonding (2:2 isomer) [from YEFRAF (Liu et al., 2014)] or **(D)** through some other linker such as 4,4′-bipyridine from Zhao et al. (2008).

Hypothetically, one can construct several potential Double Dawson structures based on common motifs of POM chemistry (Figure 5; Dawson, 1953; Zhao et al., 2008; Barrier et al., 2009): the two Dawson POM structures being connected through a joint oxygen atom on a corner (corner-shared, Figure 5A) or on an edge (edge-shared, Figure 5B) or the two Dawson units can be connected through H-bonding (Figure 5C) or some other linker (Figure 5D). With two chemically distinct tungsten sites, further complexities arise with H-bonding even, with belt-to-belt, belt-to-cap, and cap-to-cap possibilities. In the 1I94 structure, the shortest distances between the clusters 1,001 and 1,004 themselves is about 2.365 and 2.367 Å, respectively. These distances are much too long to allow consideration of an edge-shared, or even corner-shared type arrangement, even if the geometries were more reasonably oriented. These distances *are* of a length typical of a H-bond (Crans et al., 1994; Tocilj et al., 1999; Barrier et al., 2009; Bijelic and Rompel, 2015; Winkler et al., 2017). Such considerations suggest that if the clusters on the ribosome are Double Wells-Dawson Clusters, that they would be like that shown in Figure 5C and shown for the Double Cluster on the ribosome in Figure 3 (Dawson, 1953; Zhao et al., 2008; Barrier et al., 2009). Importantly, the part of the cluster in which the connection between the clusters is made is that of the cap on each cluster, which is the position of the interactions of clusters that has been observed for the Wells-Dawson Double Clusters in the literature (Figures 2, 5; reference codes JETSOR, PUPJUG, PUPJOA, and FUVXAW as well as the Wells-Dawson complex) as detailed below (Dawson, 1953; Zhao et al., 2008; Barrier et al., 2009). With this interaction of the two P_2_W_18_ clusters in mind, we may now consider the interactions with the rest of the T30S structure.

### Interactions of a Possible Double Wells-Dawson Structure (1,001···1,004) and Protein Moieties in the Small Ribosomal Subunit Structure

**Figure 6:**
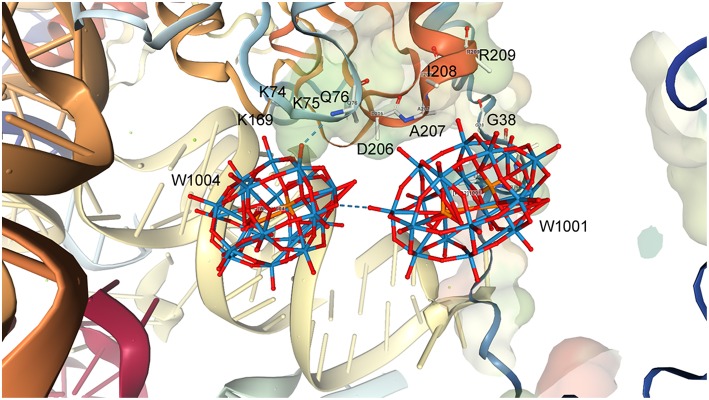
The structure of a representative Double Wells-Dawson cluster consisting of clusters 1,001 **(Right)** and 1,004 **(Left)** from 1I94.

The interactions of Clusters 1,001 and 1,004 with the ribosome structure shown in Figure 6 were identified and the distances measured and listed in Table 1. Interactions of <5 Å are considered significant with POMs in proteins (Crans et al., 1994; Felts et al., 2006; Steens et al., 2010; Goovaerts et al., 2013; Bijelic and Rompel, 2015, 2017; Arefian et al., 2017; Winkler et al., 2017), and shorter interactions (between 1.8 and 3.6 Å) can be considered possible for hydrogen bond interactions (Crans et al., 1994). The POM anions contains three different oxygen-atom sites that the protein can interact with. Which oxygen atoms associate most with the protein and the distances associated will define the stability of the complex formed between the ribosome and the POM.

**Table 1:** Interactions between Wells-Dawson Clusters 1,001 and 1,004 with protein moieties (Pioletti et al., 2001).

**Cluster**	**Residue**	**Distance, Å**	**Interaction**
1,001	Glu B 35	4.33	backbone N··· O-W_2_
1,001	Glu B 35	4.53	backbone N···O = W
1,001	Arg B 36	3.38 3.49 4.35 4.53	O···O-W_2_
1,001	Arg B 36	3.49 4.55	O···O = W
1,001	Asn B 37	4.18 4.46	backbone N··· O-W_2_
1,001	Asn B 37	4.27	O···O = W
1,001	Asn B 37	1.44 3.54 3.82 3.95	O···O-W_2_
1,001	Gly B 38	3.40 4.35	backbone N···O = W
1,001	Gly B 38	4.16 4.97	backbone N··· O-W_2_
1,001	Ala B 207	3.61 4.16	backbone N···O = W
1,001	Ala B 207	4.62 4.90	backbone N··· O-W_2_
1,001	Ile B 208	3.01 4.10	backbone N···O = W
1,001	Ile B 208	4.14 4.52	backbone N··· O-W_2_
1,001	Arg B 209	3.67	backbone N···O = W
1,001	Arg B 209	4.80	backbone N··· O-W_2_
1,004	Thr B 73	4.26	O···O = W
1,004	Lys B 74	4.09	backbone N···O = W
1,004	Lys B 75	2.09 4.90	backbone N···O = W
1,004	Lys B 75	3.92 4.39 4.43 4.93	backbone N··· O-W_2_
1,004	Gln B 76	2.96	backbone N···O = W
1,004	Gln B 76	4.01 4.86	backbone N··· O-W_2_
1,004	Lys B 169	4.87	O···O = W

As seen from Table 1, Figure 6, and Figure S2, some of the amino acids are interacting with several parts of the POM including both the bridging and W = O oxygen atoms. Table 1 shows that the Wells-Dawson clusters are mainly interacting with the peptide backbone of neutral amino acids (Ala, Gln, Glu, Ile) and with the side chains of neutral amino acids (Asn, Thr) and backbone and side chains of positively charged amino acids (Lys and Arg). In Cluster 1,001, there is an unrealistically short interaction with the oxo of Asn37 and then 27 more modest interactions with side chains and the protein backbone, with the closest being an oxo from the cluster interacting with the nitrogen atom in the backbone of Ile208. The closest interactions with Cluster 1,001 therefore come from two regions of the ribosome with amino acids 35–38 and from amino acids 207–209, forming a region of protein and POM closely associated with each other, as described previously, in a nest. For Cluster 1,004, though, there are fewer interactions than with Cluster 1,001, and most are with the nitrogen atoms on the protein backbone. The two closest interactions are between a single W = O unit and both the Lys75 and Gln76 backbone. The part of the protein that associates with the POM can be several parts of the peptide both on the backbone and on the side chains. The backbone interacting with the POM includes both C = O as well as the amide nitrogen-atom of the ribosomal subunit. In the case of both clusters, there are numerous other interactions with side chains, but all are longer than 5.0 Å. Owing to the nature of the deposited data, only interaction information with the backbone with the Wells-Dawson clusters is available. Ideally, one would prefer to have both the cluster *and* the side chain positional information.

### Precedent for Double Wells-Dawson Structures in the Solid State

A search of the Cambridge Crystal Structure Database (CSD, November 2017 update) (Allen, 2002) for crystallographically-reported Double Clusters was conducted. A general summary of the data in the CSD is presented. Using ConQuest (Bruno et al., 2002) as a searching tool, 3,752 structures containing W, P, and O all in the same molecule, 3,111 clusters in CSD contain a W—O—W unit, and 2,804 structures contain W—O—W—O—W were identified. Within those data, 952 clusters contain both W/P/O and W—O—W—O—W. If one examines all six coordinate W(= O)O_5_ functionalities in the CSD database, there are a total of 20,405 such sites which have an average W = O length of 1.711 Å (range of 1.077–2.435, with a median of 1.710 Å). The W—O average of *all* data is 2.0024 Å with a minimum of 1.077 and maximum distance of 3.612 Å, respectively. Further analysis of the tungsten coordination geometry within the group of 952 clusters mentioned above of W/P/O and W—O—W—O—W-containing clusters yield 630 compounds which have these coordination geometrical parameters and gives six coordinate W(= O)O_5_ units with an average W = O distance of 1.703 Å (with a range of 1.571–1.808 Å). The W—O average of *all* these data in the subset is 2.00099 Å with a minimum of 1.697 and a maximum W—O distance of 2.518 Å.

Of principal interest to this work was the W-systems within the Wells-Dawson cluster itself, not just general WO_6_ systems. In a P_2_W_18_O_62_ targeted search, there are 146 hits, 105 of which have coordination geometric parameters such as angles and distances. This leaves a total of 1,069 sites described as six coordinate W(= O)O_5_ in these Wells-Dawson structures. These structures have an average W = O distance of 1.703 Å (range of 1.571–1.808 Å), which is a more narrow range than the distance noted in the more general search described above. The W—O average of *all* Wells-Dawson structure data gives an average bond length of 2.001419 Å with with a minimum distance of 1.697 and a maximum distance of 2.518 Å. This is a more limited range of compounds. Because the authors based the atomic positions of the W atoms in the ribosome structure largely by using the average positions in a typical Wells-Dawson structure, the Wells-Dawson structures described in the deposited protein structures fall within these parameters.

## Discussion

The importance of POMs and their interactions with proteins has been established in the preceding sections. Here we will focus on the Wells-Dawson POM and the structure of the small ribosomal subunit and the possibility of a Double Wells-Dawson cluster forming on the small subunit of the ribosome. The use of POMs for structure solution continues for the ribosome structure as well, including the recent elucidation of an *E coli* model (Noeske et al., 2015).

### Double Wells-Dawson Clusters

In the following sections we discuss two Wells-Dawson clusters (Clusters 1,001 and 1,004, Figure 6), that are very close together leading us to refer to them as the “Double Wells-Dawson” structure on the ribosome. We are investigating the interactions of each of the two clusters with the protein and each other, as well as investigating precedent for Double Wells-Dawson clusters reported in the literature. Because these P_2_W_18_ clusters on the ribosome are only half occupied crystallographically, it is not clear from this structure alone if this Double Wells-Dawson structure is real or if the half-occupancy in model results from *on average* (over the course of all of the atoms in the entire crystal structure) half of one of the two Wells-Dawson sites are occupied (and the other may or may not be) and *on average* half of the other Wells-Dawson sites are occupied over the course of the entire structure. Such statistical electronic distribution in this case allows for a distribution of occupancies that means there could be (a) a double cluster, (b) one or the other site occupied, or (c) neither site occupied in any given site over the entire crystal. If all these possibilities were equally favorable statistically, 25% of the time there will be a Double Wells-Dawson cluster associated with the protein and no Wells-Dawson associated with the protein, and 50% of the time there would be one of the two structures associated with the protein. However, the possibility of the Double Wells-Dawson forming requires that the structure has the proper geometry, which is what has been considered above in this manuscript. To properly evaluate these possibilities one must be able to investigate the dimensions of the system and the coordinates of the entire P_2_W_18_ must be available.

### Precedent for Double Wells-Dawson Structures

In order to consider how the Wells-Dawson clusters interact in the ribosomal subunit precedent for isolated and reported P_2_W_18_ structures are investigated. Analysis of the parameters for known species in the solid-state and solution will allow us to determine if the Double Wells-Dawson structure associated with the protein in the small ribosomal subunit has a geometrically reasonable structure. Will the observed structure be similar to the description of the Dawson Wells-Cluster shown in Figure 2A, originally identified by Dawson in his 1953 description of the structure (Dawson, 1953) or similar to any of the Double Wells-Dawson structures that have been reported in the solid state or in solution since then? Such a comparison will allow us to evaluate the possibility whether the Double Wells-Dawson structure observed on the ribosome could be real. If our analysis shows that the structure has precedent between POMs in the solid-state, including the P_2_W_18_ Wells-Dawson cluster, then it is more likely that the Double Wells-Dawson Cluster on the ribosomal subunit may actually be a real. However, in addition to investigating the geometry of the cluster, we are also interested in the occupancy of the clusters associated with the ribosome. Although the P_2_W_18_ units remain in the crystals even after several rinses (Janell et al., 2001), the fact remains that soaking of multiple crystals used to solve the X-ray structures means the possibility exists of varying cluster occupancy in any given crystal, which is then averaged in the final structure. Often the occupancy of the POMs on the ribosome varies and this impacts the observed properties of the complex. That is, half occupancy of each site of a dimer is consistent with the possibility that only one site or the other may be occupied in any given unit. Electron density is important to how the cluster associates with the protein, and impacts the occupancy as well. Furthermore, the occupancy is particularly sensitive in cases where the cluster itself sits on a symmetry axis (Mauracher et al., 2014).

### Precedent for Double Wells-Dawson Structures in the Solid State

Solid-state interactions between polyoxometalates are investigated by X-ray crystallography by characterizing the intermolecular interactions which include H-bonding, stacking, and other van der Waals interactions. These interactions have been used for designing templating effects in metal-organic-framework structures (Ban et al., 1999). In Figure 5 four different possible classes of dimeric structures are shown. In examining the 105 structures emerged in the CSD “P_2_W_18_O_62_” search, four structures that can been described as a “double cluster” were identified by containing two crystallographically unique clusters in close proximity, i.e., refcodes JETSOR, PUPJUG, PUPJOA, and FUVXAW (*vide infra*) (Gong et al., 2006; Barrier et al., 2009; Yang et al., 2010). Although these reported structures also suffer from relatively large reliability (R_1_) indices in their models, the resolution is much higher for these solid-state structures than in proteins and allows a glimpse of possible examples. One of the four Double Wells-Dawsons identified, refcode JETSOR (CCDC #257590), contains very close interaction between neighboring clusters, the terminal oxo units (O_d1_-O_d1_) are only 1.054 Å apart. For reference, a distance of 1.054 Å is near an C—H or O—H bond or could be considered as a peroxo bond by coordination chemists (Gong et al., 2006). Because no precedent for a Wells-Dawson-peroxo type coordination has been reported [although a peroxodecavanadate compound has been reported (Klištincová et al., 2009)], the Double Wells-Dawson structures described here are not within the common bond lengths either, and we have not attempted to analyze this cluster further. Combined these data do provide a framework for evaluation regarding the possibility that the Double Wells-Dawson-ribosome complex is a real dimer.

The remaining three clusters identified all consist of organic bridging structures of hydrogen-bonded networks including a counterion interaction. Interactions directly between the clusters themselves are between oxo-oxo, oxo-bridged, and bridged-bridged oxygens atoms. In the structure with refcode PUPJUG (CCDC #743675, Yang et al., 2010), there are numerous interactions, but the shortest include one terminal W = O bond on the belt (O_d2_). This oxygen interacts with a triangular face of W = O on the other cluster (O_d1_, O_d1_, and O_d2_) with distances ranging from 2.818 to 3.641 Å. In addition, the interactions of that same W = O bond with the bridging oxygens on the face (O_b1_, O_b1_, and O_b3_) occur at distances of 2.978, 3.317, and 3.470 Å. Other, longer terminal-bridging (O_d_···O_b_ in both directions) interactions exist in this complex, with the longest interactions being the O_b_···O_b_ bridging-bridging interactions. One method for describing the relationship in space between the two clusters is through the angle between the horizontal mirror planes of the clusters, defined as mean planes of the center six oxygen atoms for each. In this case, that angle is 41.16°. In the double cluster of T30S, for instance, that angle is 47.96°, indicating that the axes in the T30S are slightly less parallel but that the general structure is similar.

In the structure with refcode PUPJOA, (CCDC #743674, Yang et al., 2010) there are three 4,4-bipyridine molecules directly between the clusters and, therefore fewer direct interactions. Two belt oxo units (O_d2_) of one cluster interact with one belt (O_d2_) and one cap (O_d1_) of the other. There is a 43.78° angle between horizontal mirrors. Structure refcode FUVXAW (CCDC #701467, Zhao et al., 2008) also has 4,4-bipyridine between the clusters, but only two. Two belt oxo units of one cluster (O_d2_) interact with one belt (O_d2_) and one cap (O_d1_) of the other. The clusters have a 41.47° angle between the horizontal mirrors. There are limited interactions, all quite long: terminal O_d1_ to O_d2_ of 3.000 Å and O_d2_ to O_d2_ of 3.579 Å, several terminal to bridging oxygens in both directions, ranging from 4.401 to 5.76 Å. None of these clusters are consistent with the corner- or edge-sharing POM models proposed in Figures 5A,B, but rather each is more consistent with a 2:2 or H-bond shown in Figures 5C,D.

In summary, it is noticeable that the observed interactions with the ribosome involves the oxo groups on the Wells-Dawson structures in contrast to the protein decavanadate (V_10_${\text{O}}_{28}^{\text{q}-}$) and Keggin (general form XMo_12_${\text{O}}_{40}^{\text{q}-}$) structures where the interactions often involve the bridged oxygen atoms, but akin to the Anderson-Evans (TeW_6_${\text{O}}_{24}^{6-}$) cluster, which also involves the terminal oxo atoms (Blazevic et al., 2015; Bijelic and Rompel, 2017). In the case of the Double Wells-Dawson structures of the types shown in Figure 6C the oxo groups are often paired with another oxo group bridged by, for example, H_2_O, H_3_O^+^, R_2_NH, or R_2_N${\text{H}}_{2}^{+}$ molecules, the latter illustrated in Figure 5C. Although the very first and original X-ray structure by Dawson lacks the detail to show H-bonding interactions categorized in Figure 5C (Dawson, 1953), the original structure has beautiful hydrogen bond interactions with a small cation bridging two clusters in a manner that qualify as a “Double Wells-Dawson” in the manner we are describing in this work.

Of the 105 Wells-Dawson-cluster-containing structures in the CSD, several examples of short cluster-cluster interactions with symmetry equivalent clusters are known. Exemplary structures include compounds with structure codes COSVIR (CCDC #1003304, Chen et al., 2014) and RIBFUF (CCDC #933195, Kato et al., 2013), which show belt-to-belt interactions of the oxo units, whereas GUHNIH CCDC #718729, (Kurashina et al., 2009) and YEFRAF (CCDC #653498, Liu et al., 2014) show cap-to-cap interactions. Specifically, COSVIR has short interactions of 2.662 Å between belt oxo units on adjacent clusters (O_d2_) along with several longer interactions (3.116 and 3.213 Å) and interactions between the oxo unit (O_d2_) and a bridging oxygen (O_b1_ or O_b2_) on the neighboring cluster (3.013 and 3.323 Å). Hydrogen bonding with a likely water molecule between a belt oxo (O_d2_) and a cap oxo (O_d1_; 2.968 and 2.869 Å O-H···O, respectively) is also present. RIBFUF has many interactions between the symmetry-equivalent clusters, but a 2.868 Å O_d1_···O_d1_ interaction is the shortest. The short cluster-cluster interactions in the T30S Ribosome structures is better described as cap-to-cap, and GUHNIH and YEFRAF are examples of structures of that type. As shown above, in 1I94 (Pioletti et al., 2001) the double cluster unit appears to have a cap oxo unit in one cluster (O_d1_ in 1,001) in close contact with a bridging oxo as well as two cap oxo units on the adjacent cluster (O_c_ and O_d1_, respectively in 1,004). In YEFRAF, the O_d1_ oxo unit is 2.716 Å from the other symmetry-generated oxo unit and 2.968 and 3.218 Å from O_c_ bridging atoms in the adjacent symmetrically-equivalent cluster. In GUHNIH, there are many more interactions between the two symmetry-equivalent clusters; oxo-oxo (O_d1_···O_d1_) distances are 3.577 to 4.204 Å; unique distances between the oxo units and the adjacent-cluster bridging oxygen atoms (O_c_) are 2.794–3.626 Å; whereas the unique bridging-oxygen to bridging-oxygen distances are 3.073–4.127 Å. The YEFRAF structure is furthermore stabilized by hydrogen bonding through four dimethylammonium cations between the caps of the clusters. These symmetry-generated Double Wells-Dawson clusters, then, also have inter-cluster distances that appear in line with the Double Wells-Dawson clusters seen in the ribosomal structures.

### Precedent for Double Wells-Dawson Structures in Solution

In addition to forming dimers that are characterized by X-ray crystallography, information is available on the structural evolution in polyoxometalates in solution. There are no specific examples reported of P_2_W_18_ clusters dimerizing/polymerizing in solution, but derivatives and lacunary species of these and other POMs have been examined, often leading to compounds with interesting properties. For example, the Zr(IV)–containing polyoxometalates derivatives that are found to effectively cleave proteins near aspartate residues (Absillis and Parac-Vogt, 2012; Ly et al., 2013; Stroobants et al., 2013; Vanhaecht et al., 2013). Both experimental studies and theoretical studies are available in some cases documenting the dimerization of polyoxometalates and formation of different isomers (Gong et al., 2006; Zhao et al., 2008; Barrier et al., 2009; Kurashina et al., 2009; Yang et al., 2010; Liu et al., 2014), e.g., the mechanistic study of peptide bond hydrolysis by the Wells-Dawson cluster (Absillis and Parac-Vogt, 2012). Dimerization of Lindqvist and Keggin Clusters through M-μ-O-M junctions have been investigated using DFT methods (Lopez et al., 2006), but it should be noted that W is not commonly engaged in these processes and dimer formation is more common for metal ions such as Nb, Ti, Cr, Fe, and Zr (Gong et al., 2006; Zhao et al., 2008; Barrier et al., 2009; Kurashina et al., 2009; Yang et al., 2010; Liu et al., 2014). Some of these clusters form through interaction with coordination complexes such as a peptide complex which can then react by replacement of the ligand to form a dimer (Lopez et al., 2006). Such dimers have been characterized by X-ray crystallography, and importantly information is available on the reactivity and structural changes of these polyoxometalates in solution.

Another common method of dimerization is through coordination of organometallic ligands which can react to form larger structures (Nomiya et al., 2001). Most of the organometallic complexes are covalently bound to three of the bridging oxygen atoms in a cap of the cluster unit documenting the need for the complex coordination (Edlund et al., 1988; Pohl et al., 1995; Nagata et al., 1997; Nomiya et al., 2007). The Wells-Dawson clusters and derivatives are very versatile and are known to form dimeric/polymeric structures with strong covalent bonds and interactions as hydrogen bond ones. These strategies have been used effectively for synthesis of larger structures, and in some of these derivatives the organometallic unit persists; in others the unit has been replaced. Indeed, ligands have been found to affect reactivity showing that the *cap* region of the Wells-Dawson reacts first (Poblet et al., 2003). The presence of water has even been reported to change the selectivity of the catalysts on solid heteropolyacids (Micek-Ilnicka, 2009) documenting that water molecules and potential protonation can have a dramatic effect on the reactivity and catalysis of the Wells-Dawson cluster. Wells-Dawson clusters and the protonation states have been shown to be very important in catalysis (Wang and Yang, 2015).

Because the POM cluster contains central (O_a_), terminal (O_d_), edge-sharing (O_c_), and corner-sharing (O_b_) oxygen atoms (Figure 2), there is a potential to protonate different oxygen atoms and the literature is divided on which oxygen is most basic (Lopez et al., 2002; Poblet et al., 2003). DFT calculations on X_2_M_18_${\text{O}}_{62}^{\text{q}-}$ clusters (Vilà-Nadal et al., 2012) report that the edge-sharing oxygen (O_c_) atoms are the preferred proton location sites but the stabilization is <10 kJ mol^−1^ from the other possible sites (Janik et al., 2009). These studies also report that the first unoccupied molecular orbital (LUMO) of the Wells-Dawson is delocalized over the equatorial/belt region, whereas the first virtual orbital located on the *cap* region has been computed to be 0.85 eV higher in energy (Absillis and Parac-Vogt, 2012; Vilà-Nadal et al., 2012). Since ligands and the environment of the cluster are known to impact the stability of POMs, such effects are likely to be important for the observed preferential structures.

The effects of ligands, pH, and environments may reconcile the seemingly contradictory and inconclusive literature with regard to protonation of POMs (Howarth and Jarrold, 1978; Ozeki et al., 1994; Minato et al., 2014; Lopez, 2017). Studies on decavanadate (V_10_${\text{O}}_{28}^{\text{q}-}$) and Keggin anions (general form XMo_12_${\text{O}}_{40}^{\text{q}-}$) show that the bridging oxygen atoms (O_c_ and O_b_) are the most basic sites (Roman et al., 1995; Lopez, 2017; Zhang et al., 2017), however, this is not what is observed in the Double Wells-Dawson structure (Rocchiccioli-Deltcheff et al., 1983; Minato et al., 2014; Lopez, 2017), where the terminal oxygen-atoms (O_d_) are the functionalities associating with the ribosomal protein. It is known that ligands can affect the site of protonation and probably yield a very different set of HOMO-LUMO orbitals (Lopez et al., 2002; Zhang et al., 2017). The fact that conclusions from studies based on FT-IR spectroscopy (Rocchiccioli-Deltcheff et al., 1983), ^17^O NMR spectroscopy, and various NMR spectroscopic methods may not directly confirm the theoretical predictions is also important because the DFT calculations generally focus on properties of compounds in the gas phase (Rocchiccioli-Deltcheff et al., 1983; Lopez et al., 2002; Poblet et al., 2003; Li et al., 2006; Leng et al., 2009; Vilà-Nadal et al., 2012; Minato et al., 2014; Wu et al., 2015). Therefore, the stability order may vary as observed when comparing results from gas phase calculations with experimental solution experiments (Lopez et al., 2002; Poblet et al., 2003; Lopez, 2017).

We therefore conclude that combining these observations supports the potential for coordinating oxygen sites could associate with the ribosomal subunit and that possible complexes could be sufficiently close in energy allowing for protein-POM complexes. Consequently, observation of a Wells-Dawson cluster associating with the ribosome can be assisted by protonation, hydrogen-bonding, or metal ion complexation. In solution the oxo-groups are generally protonated following the μ-O atoms, and this stabilization may explain why so many of Wells-Dawson clusters associate with the protein through the oxo groups.

### Evaluating if the Double Wells-Dawson on the Ribosome Is Real

Given the literature precedents for Double Wells-Dawson clusters, we now re-examine the Double Wells-Dawson cluster in the ribosomal subunit structure, specifically 1I94. The interactions that we have identified from the point of stabilizing the protein with more short distances to Cluster 1,001 suggest that this cluster is the most stabilizing cluster in the Double Dawson cluster. However, when considering that Cluster B is supporting the same amino acids 74–76 in both clusters, the two clusters do seem to both stabilize the ribosome structure and thus support the possibility that the Double Wells-Dawson cluster is a real dimer. As Bashan and Yonath had noted previously (Bashan and Yonath, 2008), the Wells-Dawson and Double Wells-Dawson clusters supported a rearrangement of the protein subunit that not only stabilize this conformation of the protein structure but also allows crystallization. In the Wells-Dawson-containing ribosome-X-ray structure for PDB ID 1I94 there are eight interactions <2.0 Å, and one is as close as 1.33 Å. In all these systems the interactions are otherwise chemically reasonable and the structures have normal distances with electrostatically favorable structural arrangements. However, the low occupancies in the model system presented for the highlighted clusters do allow the possibilities that each Wells-Dawson cluster in the Double Wells-Dawson cluster are not present in both sites at the same time, but the geometry and coordination environments allow for both clusters to be present.

The distances and angles between Clusters 1,001 and 1,004 in the double Double Wells-Dawson are also consistent with a real interaction (that is a normal bond/interaction) between Wells-Dawson clusters found in the solid state in the CSD. Distances are consistent with the known structures containing the Wells-Dawson cluster and are consistent with a cap-to-cap interaction of the two clusters. Solution data in conjunction with theoretical data also support the existence of Double Clusters of the type that is observed on the ribosome. The structures outlined in Figure 5 show the range of Double Clusters that can form. The cluster formed on the small ribosomal subunit appear to be one that is supported through H-bonding and illustrated in Figure 5C. The discussion of protonation and basic sites on the Wells-Dawson cluster in solution is strongly influenced by what is observed on the smaller clusters that have been more extensively investigated such as the decavanadate, Anderson-Evans, and Keggin structures. In the case of the Wells-Dawson cluster the most basic oxygen-atoms are less clear as well as the effect of environment on cluster formation. Therefore, the information in the literature would suggest that the structure formed on the ribosome would not necessarily be the isomer or conformation expected to form in solution. The observation that the Double Cluster consists of interactions through mainly the W = O units and less so the bridging oxygen atoms from the point of view of the POM and from backbone interactions from the point of view of the protein is not what would have been anticipated. However, POMs have been shown to be sensitive to their environment and that ligands can favor a behavior different than that generally observed. It is therefore possible that the presence of the protein does change the stability order of which oxygen atom is most basic and thus explains the observed form of the Double Wells-Dawson structure (2:2 isomer). As seen in Figure 6, Cluster 1,004 is interacting with Cluster 1,001 through a W = O unit and Cluster 1,001 is interacting through a W = O and a bridged oxygen atom.

### Potential Applications of Wells-Dawson-Ribosome Clusters

The presence of the POM on the ribosome has been shown to impact the structure of the ribosome and helps the organization of it within the crystals, thus leading to higher resolution diffraction and to a refinement of the crystal structure (Thygesen et al., 1996; Tocilj et al., 1999; Weinstein et al., 1999; Schluenzen et al., 2000; Auerbach-Nevo et al., 2005; Bashan and Yonath, 2008; Yonath, 2009). Highly stable and symmetric clusters are desirable for phasing [see for example (Blazevic and Rompel, 2016)], but multi-metal clusters for crystallization are often unpredictable because the interactions typically occur between high symmetry clusters and low symmetry proteins (Thygesen et al., 1996; Tocilj et al., 1999). There is also a tendency for the clusters to bind along crystallographic symmetry axes (see Ladenstein et al., 1987; Thygesen et al., 1996; Rudenko et al., 2003; Dahms et al., 2013).

The T30S ribosomal subunit structure reported previously has 28 sites for Wells-Dawson polyoxotungstates per dimeric unit (Tocilj et al., 1999; Weinstein et al., 1999; Pioletti et al., 2001; Bashan and Yonath, 2008), each of which are half-occupied (Figure 1C). In later ribosome structures the location of these polyoxometalates in the structure deposited in the Protein Data Bank (PDB) (Berman et al., 2000; Schluenzen et al., 2000) database (both 1FKA and 1I94) were simplified from the point of view of the protein. However, these coordinates do not always include the detailed Wells-Dawson structural information and thus lose information with regard to the protein-oxometalate interactions. Because of the symmetry of the space group in which the ribosome crystallizes (*P*4_1_2_1_2), 14 of these sites are unique as described subsequently.

One of the more surprising results in the studies is that the shortest bond lengths and interactions are with the nitrogen-backbone residues rather than side chains, although much side chain information is not detailed in the deposited data. Most interactions are also with the terminal oxo units (O_d_) on the Dawson clusters, not the bridging oxygen atoms (O_b_ or O_c_), which is surprising given that the most basic sites and hydrogen bonding acceptor is more likely the bridging oxygen-atoms (*vide supra*; Lopez et al., 2002; Poblet et al., 2003; Janik et al., 2009). In general, these interactions are therefore not what one would have expected both from the point of view of the POMs and from the point of view of the protein. Further analysis of a more complete data set is desirable.

The fact that the Wells-Dawson-ribosome interface was made up not by one single part of the protein, but that amino acids came from different parts of the peptide show that the protein is folding and packing in the presence of the POM (Figure 4C). Similar reorganization of the peptide structure has also been reported for the tyrosinase enzyme (Mauracher et al., 2014) and demonstrates that these types of interactions are important not only for the interactions with the ribosomal protein but in a more general sense as well (Tocilj et al., 1999). The ability of POMs, and specifically P_2_W_18_, to template interactions with surrounding peptide ligands result in stable POM-protein complexes that can facilitate protein crystallization.

## Conclusions

In this manuscript, we have characterized the molecular details of the Wells-Dawson Clusters (P_2_W_18_) associated with the ribosome as well as one Double Wells-Dawson cluster using data mining. We have examined the interactions of the P_2_W_18_ with the small ribosomal subunit, including the interactions of a pair of two Wells-Dawson structures close together. It was found that the stabilization of the ribosome appears to be mainly through interactions of the peptide backbone with the W = O groups in the P_2_W_18_ clusters and by the side chains of positively charged amino acids Lys and Arg. By examining the reported examples of Double Clusters we found that the Double Cluster on the ribosome has a structure consistent with the reports in the literature of Double Wells-Dawson clusters in the solid state and in solution. However, it appears that the isomers formed in aqueous solution are different than the form we observe on the ribosomal subunit in a less polar environment. We conclude that the data obtained are consistent with the Double Cluster formed on the ribosome being structurally possible and a real structure. These results and current data sets do not preclude the possibility that some of the structures have partial occupation of each of the two separate sites.

## Data Availability

All datasets generated for this study are included in the manuscript and/or the Supplementary Files or are available through the PDB /CSD.

## Author Contributions

DC and CM wrote on the manuscript and worked on the project in a collaborative manner, although the expertise tended to divide the contributions in the areas of the biology and data interpretation (for DC) and crystallography (for CM). IS-L carried out the literature search for the solution chemistry of Wells-Dawson complexes.

### Conflict of Interest Statement

The authors declare that the research was conducted in the absence of any commercial or financial relationships that could be construed as a potential conflict of interest.
