# Supplementary material for: Exploring Wells-Dawson Clusters Associated With the Small Ribosomal Subunit
Source: Front Chem. 2019 Jul 5;7:462. doi: 10.3389/fchem.2019.00462 (PMC6624422; doi:10.3389/fchem.2019.00462)
Supplement: Supplementary file 1 [file Data_Sheet_1.pdf]

Exploring Wells-Dawson Clusters associated with the Small Ribosome Subunit

Supporting Information

Debbie C. Crans,[1*] **Irma Sánchez-Lombardo**[1,2] **and Craig C. McLauchlan**[3*]
[1] Department of Chemistry, Colorado State University, Fort Collins, 80523 USA Fax: +970-491-7635 E-mail: Debbie.Crans@ColoState.edu http://wp.natsci.colostate.edu/crans/

[2] División de Ciencias Básicas, Universidad Juárez Autónoma de Tabasco, Cunduacán, Tabasco 86690, Mexico; Email: irmas_l@yahoo.com.mx

[3] Department of Chemistry, Illinois State University, Normal, IL 61790-4160 USA Fax: +309-438-5538 E-mail:McLauchlan@IllinoisState.edu http://chemistry.illinoisstate.edu/ccmclau/

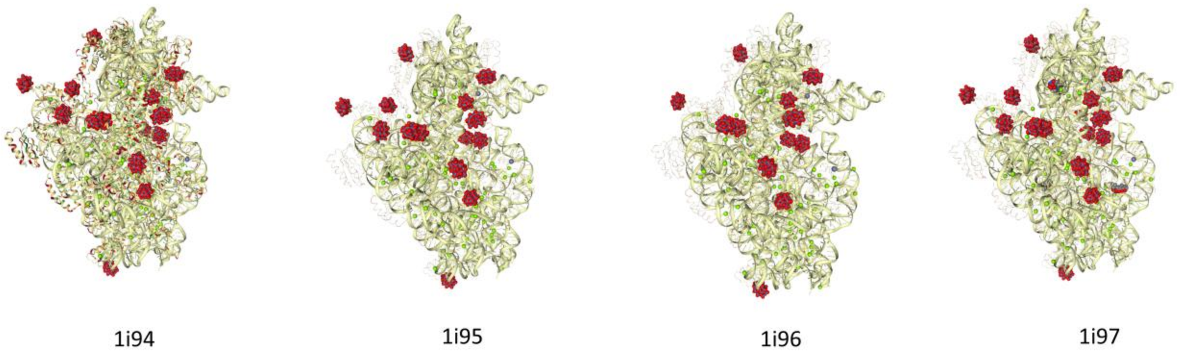

Figure S1. Comparison of ribosome structures from Protein Data Bank showing P_2W_{18} clusters (red).

Table S1. All WO2 –containing structures from Protein Data Bank (PDB).

PDB Code	Structure	Reference
1I94	T30S Ribosomal subunit at 3.2 Å resolution	Pioletti *et al.*, 2001
1I95	T30S Ribosomal subunit with Edeine	Pioletti *et al.*, 2001
1I96	T30S Ribosomal subunit with IF3	Pioletti *et al.*, 2001
1I97	T30S Ribosomal subunit with tetracycline	Pioletti *et al.*, 2001
1FKA	T30S Ribosomal subunit at 3.3 Å resolution	Schluenzen *et al.*, 2000
1DV4	Partial structure of 16S RNA of T30S	Tocilj *et al.*, 1999

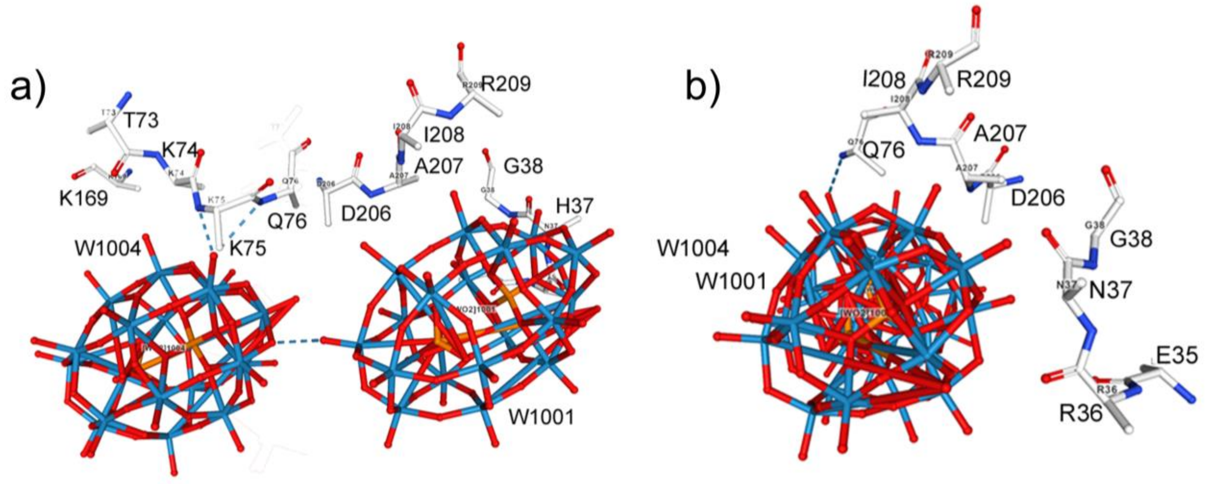

Figure S2. Alternative views of Figure 5 showing more detail of interactions between representative Double Wells-Dawson cluster consisting of clusters 1001 and 1004 with protein in 1i94 a) as shown in Figure 5, and b) rotated by ~90° to show interactions with residues 35-38.
